# Association between urinary heavy metals and cardiovascular-kidney-metabolic syndrome: mediating roles of TyG, WWI, and eGFR

**DOI:** 10.3389/fnut.2025.1613721

**Published:** 2025-06-25

**Authors:** Jianping Liu, Yuanying Song, Rong Luo, Sufang Wang, Pinglei Pan, Lijian Han

**Affiliations:** ^1^Department of Neurology, Yancheng Third People’s Hospital (The Sixth Affiliated Hospital of Nantong University, The Yancheng School of Clinical Medicine of Nanjing Medical University, The Affiliated Hospital of Jiangsu Vocational College of Medicine), Yancheng, Jiangsu, China; ^2^Department of Central Laboratory, The Yancheng School of Clinical Medicine of Nanjing Medical University (Yancheng Third People’s Hospital), Yancheng, Jiangsu, China; ^3^School of Pharmacy, Nanjing Medical University, Nanjing, Jiangsu, China

**Keywords:** heavy metals, cardiovascular-kidney-metabolic syndrome, mediation analysis, NHANES, Bayesian kernel machine regression

## Abstract

**Background:**

Cardiovascular-kidney-metabolic (CKM) syndrome is a multistage disorder with significant global health and socioeconomic impact. Environmental factors are increasingly recognized as key risk factors in disease development. This study aims to assess the relationship between urinary heavy metals and CKM risk, as well as the mediating roles of triglyceride-glucose index (TyG), weight-adjusted waist index (WWI), and estimated glomerular filtration rate (eGFR).

**Methods:**

This cross-sectional analysis uses data from the National Health and Nutrition Examination Survey (NHANES). CKM stages 0–2 are classified as non-advanced, and stages 3–4 as advanced. Multivariable logistic regression, restricted cubic splines (RCS), weighted quantile sum (WQS) regression, quantile-based g-computation (Qgcomp) regression, and Bayesian kernel machine regression (BKMR) were applied to explore associations between metal exposure and advanced CKM. Mediation analysis examined the roles of TyG, WWI, and eGFR in heavy metal-induced advanced CKM.

**Results:**

Among 5,221 participants, 4,340 were non-advanced and 881 had advanced CKM. Both individual and mixed heavy metal exposures were positively linked to advanced CKM risk. Cobalt (Co) was identified as a primary contributor. TyG, WWI, and eGFR partially mediate the relationship between heavy metal exposure and advanced CKM prevalence.

**Conclusion:**

Heavy metal exposure is associated with increased CKM risk. TyG, WWI, and eGFR were found to partially mediate the association between heavy metal exposure and advanced CKM prevalence, suggesting potential pathways linking environmental exposures to CKM risk.

## 1 Background

Cardiovascular-kidney-metabolic (CKM) syndrome is a recently defined clinical syndrome that integrates the interactions between cardiovascular disease (CVD), chronic kidney disease (CKD), and metabolic disorders such as diabetes and obesity. The American Heart Association (AHA) has proposed a staging system for CKM, ranging from stage 0 (no risk factors) to stage 4 (established CVD with or without renal failure), emphasizing the progressive nature of the syndrome, with stages 3 and 4 defined as advanced CKM (1). Stage 3 of CKM syndrome is marked by pronounced metabolic disturbances, including dyslipidemia, hyperglycemia, and hypertension, along with renal impairment and increased cardiovascular strain. As the condition advances to stage 4, cardiovascular issues progressively emerge, indicating further deterioration and representing the most critical phase of CKM syndrome. Epidemiological data show that around 90% of U.S. adults meet the criteria for at least stage 1 CKM, with 15% experiencing advanced stages (2). At present, treatment options for advanced CKM remain limited, placing significant pressure on healthcare systems, partly due to an incomplete understanding of its complex etiology (3). Growing evidence highlights that environmental factors may contribute to the progression of CKM by triggering systemic inflammation, oxidative stress, and endothelial dysfunction (4, 5). Therefore, a thorough understanding of how environmental exposures influence the pathogenesis of advanced CKM is essential for the development of targeted preventive strategies.

Heavy metals are widespread environmental pollutants that enter the human body through the food chain, air, and water, accumulating within tissues and exerting multiple toxic effects on the cardiovascular, renal, and metabolic systems. In the cardiovascular system, exposure to heavy metals is closely associated with hypertension, atherosclerosis, and heart failure (6). For instance, lead (Pb) exacerbates cardiac damage by promoting oxidative stress and myocardial fibrosis (7), while maternal exposure to cadmium (Cd) is a significant risk factor for chronic heart failure in offspring (8). In the kidneys, heavy metals accumulate in the renal tubules, leading to tubule damage and a decline in glomerular filtration rate, thereby accelerating the progression of CKD (9). In terms of metabolism, barium (Ba) and Cd affect the development of metabolic syndrome through interactions with insulin and estrogen receptors (10). These studies suggest that heavy metals may be key environmental factors linking cardiovascular, renal, and metabolic pathologies.

In the pathophysiology of CKM syndrome, the triglyceride-glucose index (TyG), weight-adjusted waist index (WWI), and estimated glomerular filtration rate (eGFR) are three key biomarkers. TyG, as an alternative indicator of insulin resistance, has been associated with an increased prevalence of advanced CKM as the index rises (11). WWI reflects visceral fat accumulation and is independently correlated with arteriosclerosis and proteinuria, serving as an important link between obesity and cardiovascular-renal outcomes (12, 13). eGFR is a core indicator for assessing kidney function, and its decline is closely associated with increased risks of CVD, end-stage renal disease (ESRD), and all-cause mortality, making it a critical marker for advanced CKM progression (14). It is crucial that heavy metals may disrupt these biomarkers: the TyG index shows an upward trend with the increase in the concentrations of Pb and Cd in the blood (15), while higher blood manganese levels are positively correlated with poor visceral fat tissue quality and an increased risk of visceral obesity (16). Moreover, Cd exposure directly accelerates the decline in eGFR (9). Therefore, studying the mediating roles of TyG, WWI, and eGFR in the relationship between urinary heavy metals and advanced CKM will not only help uncover the potential mechanisms of heavy metal exposure in advanced CKM but also provide new insights for early prevention and intervention strategies.

This study performed a cross-sectional analysis using the 2005–2018 National Health and Nutrition Examination Survey (NHANES) database to investigate the association between nine urinary metals and advanced CKM risk, while also assessing the mediating roles of TyG, WWI, and eGFR.

## 2 Methods

### 2.1 Study population

**FIGURE 1:**
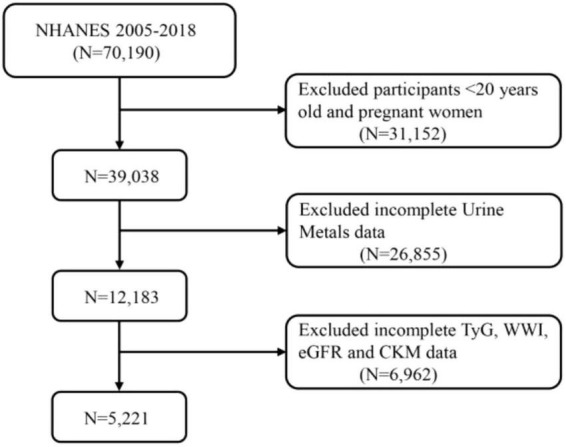
A flow diagram of eligible participant selection in the National Health and Nutrition Examination Survey. TyG, triglyceride-glucose index; WWI, weight-adjusted waist index; eGFR, estimated glomerular filtration rate; CKM, cardiovascular-kidney-metabolic syndrome.

This study included participants from the 2005 to 2018 NHANES database. NHANES is a cross-sectional survey conducted by the National Center for Health Statistics (NCHS) aimed at collecting health and nutrition information from a non-institutionalized U.S. population. Figure 1 illustrates the data selection process used in our study. All procedures were approved by the NCHS Ethics Review Board, and written consent was obtained from all participants. Among the 70,190 participants, individuals under 20 years of age and pregnant women (*n* = 31,152), as well as those missing data on heavy metals (*n* = 26,855), TyG, WWI, eGFR, and CKD (*n* = 6,962), were excluded. Ultimately, the study included 5,221 participants with complete information.

### 2.2 CKM syndrome assessment

According to the pathophysiological mechanisms, prevention-treatment strategies, and disease risk, CKM syndrome is classified into five clinical stages (1): stage 0 (no risk factors): all conditions are normal; stage 1 (metabolic precursor phase): simple obesity or prediabetic state; stage 2 (metabolic disorder phase): at least one other metabolic abnormality or CKD present; stage 3 (subclinical cardiovascular phase): metabolic abnormalities or CKD combined with subclinical CVD; stage 4 (clinical cardiovascular phase): clear clinical manifestations of CVD in patients with metabolic abnormalities or CKD. CKM prevalence was quantified using weighted estimates to represent the U.S. population, based on NHANES sampling weights (WTSAF2YR, adjusted for 2005–2018). Diagnostic criteria followed the AHA’s CKM staging framework (1), with detailed criteria provided in Supplementary Table 1. Stages 0–2 were classified as non-advanced CKM, and stages 3–4 as advanced CKM, reflecting increasing severity of metabolic, renal, and cardiovascular impairments. Unless otherwise specified, CKM referred to as advanced CKM in all results except Result 1.

### 2.3 Metal measurement

The urinary metal data were sourced from NHANES 2005–2018. Ba, Cd, cobalt (Co), cesium (Cs), molybdenum (Mo), Pb, antimony (Sb), thallium (Tl), and tungsten (W) were primarily measured using inductively coupled plasma mass spectrometry (ICP-MS). Values below the limit of detection (LOD) were substituted with half of the square root of the LOD. Metal concentrations were adjusted for urinary creatinine and presented as micrograms per gram of creatinine.

The nine urinary metals (Ba, Cd, Co, Cs, Mo, Pb, Sb, Tl, and W) were selected due to their documented associations with cardiovascular, renal, and metabolic toxicities in prior literature, as well as their consistent measurement in the NHANES 2005–2018 dataset using ICP-MS, which ensures analytical reliability.

### 2.4 Covariates and mediators

This study considers covariates such as age, sex, race, education level, marital status, family poverty income ratio (PIR), obesity, smoking status, alcohol consumption, diabetes, hypertension, and hyperlipidemia. Additional details on these covariates are provided in Supplementary Table 2.

In this study, TyG, WWI, and eGFR were selected as mediators based on the characteristics of CKM syndrome. The TyG index is used to quantitatively assess insulin resistance by combining fasting blood glucose and triglyceride levels (17). The calculation formula is as follows:

TyG index = Ln [fasting TG (mg/dL) × fasting blood-glucose (mg/dL) / 2]

Triglyceride and fasting blood glucose levels were measured using an automated biochemical analyzer with enzymatic techniques. Serum triglyceride concentrations were determined with the Roche Cobas 6000 chemistry analyzer and the Roche Modular P system.

Weight-adjusted waist index is designed to more accurately assess central obesity by adjusting waist circumference for body weight (12). The calculation formula is as follows:


$$\mathrm{WWI}\,\left(\mathrm{cm}/\sqrt{k}\,g\right)=\mathrm{Waist}\,\mathrm{circumference}/\sqrt{W}\,\mathrm{eight}$$

Estimated glomerular filtration rate was calculated using the 2009 CKD-EPI equation, consistent with NHANES protocols during the 2005–2018 period and prior epidemiological studies (18). This equation was chosen for its widespread use and validation in large-scale studies at the time of analysis.

### 2.5 Statistical analysis

To ensure that the data represent the national population, all analyses were conducted using sampling weights. The weighted variable used in our study was the fasting subsample 2-year weight (WTSAF2YR), and the new weights for the period 2005–2018 were calculated as WTSAF2YR × 1/7. Continuous variables are presented as mean (SD), while categorical variables are shown as frequency (percentage). Weighted *t* tests were used for comparing continuous variables, and weighted Chi-square tests were applied to categorical variables. Outliers in urinary metals, TyG, WWI, and eGFR were identified using the interquartile range (IQR) method, with values beyond 1.5 × IQR below Q1 or above Q3 flagged as potential outliers. Distributions were visually inspected using rain cloud chart (Supplementary Figure 1). To ensure analytical robustness, outliers were retained in the primary analysis, with log-transformation applied to metals to approximate normality. Multivariable logistic regression was applied to estimate the odds ratios (ORs) and corresponding 95% confidence intervals (CIs) for the association between metals, mediators, and advanced CKM. Multivariable linear regression was used to explore the association between metals and mediators. Restricted cubic splines (RCS) with knots at the 10th, 50th, and 90th percentiles of the independent variable distribution were employed to assess the linearity assumption between the independent variables and the outcome probabilities, as well as to explore potential non-linear relationships (19). Subgroup analyses were performed based on age, sex, race, education, marital status, PIR, obesity, smoking, alcohol use, diabetes, hypertension, and hyperlipidemia. Spearman’s correlation was used to assess the relationship between Ln-transformed metals.

Additionally, weighted quantile sum (WQS) regression, quantile-based g-computation (Qgcomp) regression, and Bayesian kernel machine regression (BKMR) models were employed to assess the association between metal mixtures and advanced CKM. The WQS model merges multiple chemical effects into a mixture index using quantile scoring and weighting, enabling the evaluation of its relationship with specific outcomes (19). It used 10,000 bootstrap samples to ensure stable estimates. Qgcomp analysis enhances WQS regression by incorporating g-computation, maintaining WQS’s simplicity and computational ease while avoiding the assumption of homogeneous exposure-outcome relationships (20). BKMR is a new strategy that filters variables using the Markov Chain Monte Carlo algorithm and constructs Gaussian kernel functions. It is a non-parametric Bayesian variable selection framework that combines Bayesian and statistical learning methods to address potential non-linearity and non-additivity (21). All models iterated 10,000 times. Mediation analysis employed a quasi-Bayesian Monte Carlo method with 1,000 simulations using a normal approximation. The direct effect shows the impact of metal exposure on advanced CKM, while the indirect effect captures its influence through mediators. The mediation proportion was calculated by dividing the indirect effect by the total effect (22).

All statistical analyses were performed using R software (version 4.3.2). The WQS (version 3.0.5), “qgcomp” (version 2.15.2), and “bkmr” (version 0.2.2) packages were used for the WQS, qgcomp, and BKMR models, respectively. A significance level of *p* < 0.05 (two-tailed test) was used to define statistical significance.

## 3 Results

### 3.1 Baseline characteristics of study samples

Table 1 presents the baseline characteristics of 5,221 CKM patients, including 4,340 nonadvanced-CKM patients and 881 advanced-CKM patients, which collectively represent approximately 31 million CKM patients in the United States. Statistically significant differences were observed between advanced-CKM and nonadvanced-CKM patients in terms of age, gender, race, marital status, education level, income, smoking, alcohol consumption, hypertension, diabetes, and dyslipidemia (*p* < 0.05). Furthermore, compared to the nonadvanced-CKM group, the advanced-CKM group exhibited higher TyG and WWI, while eGFR was lower. Baseline characteristics stratified by CKM syndrome stage are detailed in Supplementary Table 3.

**TABLE 1:** Baseline characteristics of all participants were stratified by CKM, weighted.

Characteristic	Overall, *N* = 31,065,216 (100%)	Nonadvanced-CKM, *N* = 27,387,059 (88%)	Advanced CKM, *N* = 3,678,157 (12%)	*p*-Value
No. of participants in the sample	5,221	4,340	881	–
Age (%)				<0.001
20–40	11,690,744 (38%)	11,543,648 (42%)	147,096 (4.0%)	
41–60	11,974,829 (39%)	11,209,403 (41%)	765,426 (21%)	
>60	7,399,643 (24%)	4,634,008 (17%)	2,765,635 (75%)	
Sex (%)				<0.001
Female	15,410,293 (50%)	13,927,228 (51%)	1,483,065 (40%)	
Male	15,654,923 (50%)	13,459,831 (49%)	2,195,093 (60%)	
Race (%)				<0.001
Non-Hispanic White	20,980,865 (68%)	18,259,929 (67%)	2,720,937 (74%)	
Other	4,042,763 (13%)	3,703,173 (14%)	339,590 (9.2%)	
Non-Hispanic Black	3,217,873 (10%)	2,802,558 (10%)	415,315 (11%)	
Mexican American	2,823,714 (9.1%)	2,621,399 (9.6%)	202,315 (5.5%)	
Married/live with partner (%)				0.158
No	10,948,604 (35%)	9,543,070 (35%)	1,405,534 (38%)	
Yes	20,112,208 (65%)	17,841,003 (65%)	2,271,205 (62%)	
Education level (%)				<0.001
Below high school	5,159,568 (17%)	4,175,971 (15%)	983,597 (27%)	
High School or above	25,899,476 (83%)	23,209,382 (85%)	2,690,095 (73%)	
PIR (%)				<0.001
Poor	6,033,864 (21%)	5,047,747 (20%)	986,116 (29%)	
Not poor	22,987,914 (79%)	20,581,137 (80%)	2,406,777 (71%)	
Obesity (%)				0.089
No	19,902,198 (64%)	17,676,323 (65%)	2,225,875 (61%)	
Yes	11,146,166 (36%)	9,697,044 (35%)	1,449,121 (39%)	
Smoking (%)				<0.001
Never	16,868,950 (54%)	15,421,168 (56%)	1,447,781 (39%)	
Former	8,002,121 (26%)	6,416,880 (23%)	1,585,241 (43%)	
Current	6,191,046 (20%)	5,545,911 (20%)	645,135 (18%)	
Drinking (%)				<0.001
Never	2,905,745 (10%)	2,439,577 (9.6%)	466,168 (14%)	
Former	3,525,297 (12%)	2,702,369 (11%)	822,928 (25%)	
Mild	10,917,403 (38%)	9,615,734 (38%)	1,301,668 (39%)	
Moderate	5,031,520 (18%)	4,729,794 (19%)	301,726 (9.1%)	
Heavy	6,227,108 (22%)	5,814,837 (23%)	412,271 (12%)	
Hypertension (%)				<0.001
No	19,640,523 (63%)	18,615,172 (68%)	1,025,351 (28%)	
Yes	11,424,693 (37%)	8,771,887 (32%)	2,652,806 (72%)	
Diabetes (%)				<0.001
No	26,119,834 (84%)	23,994,886 (88%)	2,124,948 (58%)	
Yes	4,945,382 (16%)	3,392,173 (12%)	1,553,209 (42%)	
Hyperlipidemia (%)				<0.001
No	9,369,135 (30%)	8,844,052 (32%)	525,083 (14%)	
Yes	21,696,081 (70%)	18,543,007 (68%)	3,153,074 (86%)	
TyG [mean (SD)]	8.58 (0.67)	8.55 (0.66)	8.80 (0.73)	<0.001
WWI [mean (SD)]	10.94 (0.81)	10.86 (0.79)	11.53 (0.74)	<0.001
eGFR [mean (SD)]	95.89 (21.91)	98.88 (19.87)	73.61 (23.52)	<0.001

Continuous: mean (SD), *p* values via weighted *t* test. Categorical: weighted *N* (%), *p* values from weighted Chi-square. PIR, poverty income ratio; TyG, Triglyceride-glucose index; WWI, weight-adjusted waist index; eGFR, estimated glomerular filtration rate; CKM, cardiovascular-kidney-metabolic syndrome.

Supplementary Table 4 further reveals that the detection rates of most urinary metals are >95%, while the detection rate of Sb is 80.253%. Spearman correlation analysis indicated correlations between the nine urinary metals, with moderate correlations observed between Pb and Cd (*r* = 0.42) and Tl and Cs (*r* = 0.59) (Supplementary Figure 2).

### 3.2 Association between urine heavy metals and CKM

In the model 3, a one logarithmic unit increase in Cd, Co, Pb, and Sb was associated with a 1.38 (95% CI: 1.11, 1.70), 1.35 (95% CI: 1.14, 1.61), 1.23 (95% CI: 1.01, 1.50), and 1.22 (95% CI: 1.01, 1.46) times higher prevalence of CKM, respectively. When these metals were categorized into quartiles, with Q1 as the reference, the CKM prevalence in the Q4 group increased by 1.28 (95% CI: 0.74, 2.23), 2.16 (95% CI: 1.50, 3.12), 1.61 (95% CI: 0.99, 2.64), and 1.70 (95% CI: 1.14, 2.55) times, respectively. *p* for trend values were 0.08, <0.001, 0.02, and 0.01, respectively (Table 2).

**TABLE 2:** Association between urinary metals and CKM.

Urine metals (log-μg/g creatinine)	Model 1 [OR (95% CI)]	*p*-Value	Model 2 [OR (95% CI)]	*p*-Value	Model 3 [OR (95% CI)]	*p*-Value
**Ba**						
Continuous	0.87 (0.76, 1.00)	0.051	0.89 (0.77, 1.03)	0.106	0.95 (0.81, 1.11)	0.521
Q1	Reference		Reference		Reference	
Q2	0.62 (0.48, 0.79)	<0.001	0.76 (0.56, 1.03)	0.08	0.91 (0.63, 1.31)	0.62
Q3	0.66 (0.50, 0.89)	0.006	0.75 (0.51, 1.09)	0.12	0.92 (0.61, 1.39)	0.69
Q4	0.77 (0.58, 1.01)	0.060	0.79 (0.55, 1.13)	0.2	0.90 (0.62, 1.32)	0.59
*p* for trend	0.101		0.220		0.630	
**Cd**						
Continuous	1.96 (1.75, 2.20)	<0.0001	1.29 (1.10, 1.51)	0.002	1.38 (1.11, 1.70)	0.004
Q1	Reference		Reference		Reference	
Q2	1.61 (1.10, 2.34)	0.014	0.70 (0.42, 1.14)	0.148	0.75 (0.42, 1.33)	0.317
Q3	2.75 (1.98, 3.81)	<0.001	0.90 (0.57, 1.42)	0.652	0.98 (0.60, 1.58)	0.924
Q4	4.38 (3.14, 6.11)	<0.001	1.11 (0.69, 1.78)	0.661	1.28 (0.74, 2.23)	0.376
*p* for trend	<0.001		0.082		0.077	
**Co**						
Continuous	1.45 (1.26, 1.66)	<0.001	1.34 (1.14, 1.59)	<0.001	1.35 (1.14, 1.61)	<0.001
Q1	Reference		Reference		Reference	
Q2	1.17 (0.91, 1.50)	0.207	1.28 (0.95, 1.72)	0.106	1.26 (0.90, 1.76)	0.177
Q3	1.40 (1.05, 1.86)	0.023	1.43 (1.02, 2.01)	0.038	1.55 (1.05, 2.29)	0.028
Q4	1.93 (1.48, 2.52)	<0.001	2.09 (1.48, 2.95)	<0.001	2.16 (1.50, 3.12)	<0.001
*p* for trend	<0.001		<0.001		<0.001	
**Cs**						
Continuous	1.15 (0.98, 1.35)	0.088	0.76 (0.58, 1.00)	0.049	0.84 (0.63, 1.13)	0.249
Q1	Reference		Reference		Reference	
Q2	1.27 (0.96, 1.70)	0.099	0.92 (0.64, 1.32)	0.645	0.93 (0.61, 1.41)	0.723
Q3	1.52 (1.13, 2.04)	0.006	1.04 (0.71, 1.54)	0.824	1.03 (0.68, 1.57)	0.880
Q4	1.30 (0.99, 1.71)	0.058	0.78 (0.54, 1.14)	0.200	0.84 (0.55, 1.28)	0.413
*p* for trend	0.023		0.286		0.513	
**Mo**						
Continuous	1.11 (0.92, 1.34)	0.284	1.02 (0.83, 1.27)	0.820	1.05 (0.86, 1.30)	0.608
Q1	Reference		Reference		Reference	
Q2	0.92 (0.69, 1.24)	0.591	0.86 (0.59, 1.26)	0.447	0.88 (0.60, 1.30)	0.524
Q3	0.98 (0.76, 1.27)	0.865	0.87 (0.62, 1.21)	0.403	1.03 (0.73, 1.46)	0.875
Q4	1.14 (0.85, 1.53)	0.389	0.97 (0.68, 1.38)	0.864	0.97 (0.68, 1.39)	0.882
*p* for trend	0.328		0.906		0.896	
**Pb**						
Continuous	1.86 (1.63, 2.12)	<0.001	1.10 (0.92, 1.32)	0.270	1.23 (1.01, 1.50)	0.036
Q1	Reference		Reference		Reference	
Q2	1.61 (1.10, 2.34)	0.014	0.92 (0.61, 1.40)	0.699	1.17 (0.70, 1.95)	0.557
Q3	2.70 (2.01, 3.62)	<0.001	1.14 (0.79, 1.64)	0.477	1.43 (0.89, 2.30)	0.133
Q4	3.74 (2.75, 5.09)	<0.001	1.20 (0.81, 1.77)	0.361	1.61 (0.99, 2.64)	0.056
*p* for trend	<0.001		0.125		0.017	
**Sb**						
Continuous	1.10 (0.94, 1.28)	0.231	1.15 (0.97, 1.38)	0.111	1.22 (1.01, 1.46)	0.036
Q1	Reference		Reference		Reference	
Q2	1.30 (0.96, 1.77)	0.094	1.36 (0.92, 2.03)	0.125	1.34 (0.84, 2.11)	0.214
Q3	1.43 (1.06, 1.91)	0.018	1.51 (1.04, 2.19)	0.029	1.47 (0.97, 2.25)	0.071
Q4	1.31 (0.94, 1.84)	0.110	1.57 (1.07, 2.28)	0.021	1.70 (1.14, 2.55)	0.011
*p* for trend	0.090		0.017		0.008	
**Tl**						
Continuous	0.60 (0.49, 0.73)	<0.001	0.79 (0.60, 1.03)	0.077	0.86 (0.65, 1.15)	0.316
Q1	Reference		Reference		Reference	
Q2	0.66 (0.51, 0.85)	0.001	0.82 (0.60, 1.14)	0.233	0.88 (0.63, 1.24)	0.462
Q3	0.48 (0.37, 0.62)	<0.001	0.67 (0.47, 0.95)	0.026	0.75 (0.52, 1.09)	0.127
Q4	0.51 (0.38, 0.68)	<0.001	0.76 (0.50, 1.15)	0.193	0.89 (0.56, 1.40)	0.607
*p* for trend	<0.001		0.104		0.431	
**W**						
Continuous	1.13 (1.01, 1.26)	0.029	1.13 (0.98, 1.29)	0.090	1.11 (0.96, 1.28)	0.166
Q1	Reference		Reference		Reference	
Q2	1.12 (0.83, 1.52)	0.447	1.15 (0.80, 1.66)	0.443	1.14 (0.76, 1.71)	0.516
Q3	1.25 (0.91, 1.73)	0.169	1.28 (0.84, 1.94)	0.250	1.23 (0.79, 1.90)	0.349
Q4	1.32 (0.98, 1.76)	0.064	1.33 (0.93, 1.90)	0.113	1.24 (0.85, 1.81)	0.268
*p* for trend	0.047		0.106		0.256	

Model 1: no covariates were adjusted. Model 2: age, sex, education level, marital, PIR, and race were adjusted. Model 3: age, sex, education level, marital status, PIR, race, obesity, smoking, drinking, hypertension, diabetes, and hyperlipidemia were adjusted. All metals were Ln-transformed before analysis. Ba, barium; Cd, cadmium; Co, cobalt; Cs, cesium; Mo, molybdenum; Pb, lead; Sb, antimony; Tl, thallium; W, tungsten; CKM, cardiovascular-kidney-metabolic syndrome; OR, odds ratio; CI, confidence interval.

Restricted cubic splines analysis further demonstrated that after adjusting for relevant variables, there was a significant linear positive correlation between the logarithmic transformed concentrations of Cd, Co, Pb, and W in urine and the prevalence of CKM (Supplementary Figure 3). Additionally, in the WQS model, the metals with the highest weights were Co (37%), W (24%), Cd (22%), and Sb (14%) (Supplementary Figure 4). The results from qgcomp were similar to those of the WQS model, with Co contributing the most to the prevalence of CKM (Supplementary Figure 5). Subgroup analysis revealed a significant positive correlation between the concentrations of Cd, Co, and Pb in urine and CKM prevalence across all subgroups. Moreover, significant interactions were found between urinary Co and smoking status, as well as between urinary Pb and race (*p* < 0.05) (Supplementary Figure 6).

### 3.3 Heavy metals exposure and CKM risk in the BKMR model

**FIGURE 2:**
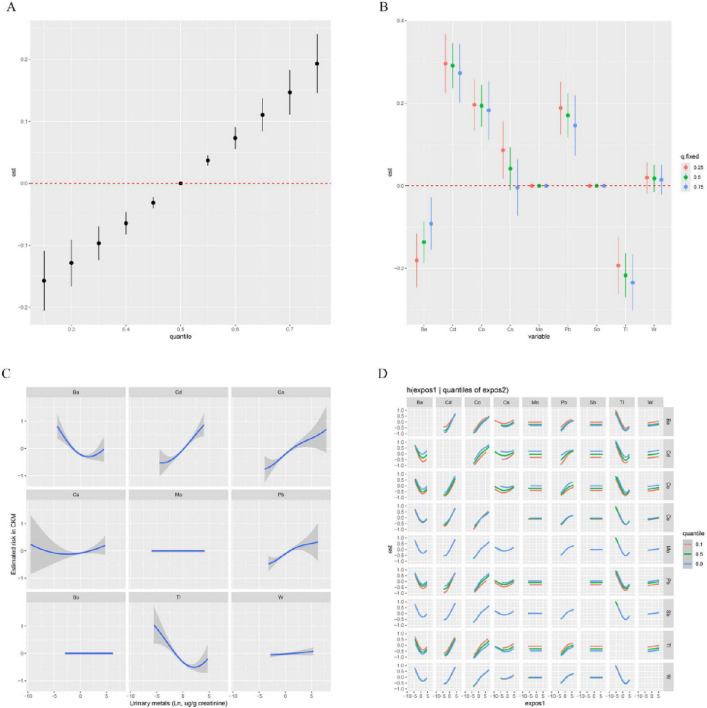
Associations of urinary metals with CKM risk estimated by Bayesian Kernel Machine Regression (BKMR). **(A)** Combined effects of urinary metals mixture on CKM. **(B)** Single-exposure CKM effects (95% CI), defined as the change in the response associated with a change in a particular exposure from its 25th to its 75th quantile, where all of the other exposures are fixed at a specific quantile (0.25, 0.50, or 0.75). **(C)** Exposure-response functions for each metal with the other metals fixed at the median. **(D)** Bivariate exposure–response function of metals with CKM: investigating exposure–response function with varying quantiles of second exposure, while other exposures are fixed. Orange, green, and blue are the 0.1, 0.5, and 0.9 quantile of the second exposure. All metals were Ln-transformed before analysis. Models were adjusted for age, sex, education level, marital status, PIR, race, obesity, smoking, drinking, hypertension, diabetes, and hyperlipidemia.

We further employed BKMR to estimate the association between the mixture of metals in urine and CKM risk. The results indicated a significant positive correlation between the mixed metals and CKM risk (Figure 2A). Additionally, when controlling for other metals at the 25%, 50%, and 75% levels, the levels of Cd, Co, and Pb in urine showed a positive effect on CKM prevalence (Figure 2B). Figure 2C shows that when the concentrations of all other heavy metals were held at the median, the levels of Cd, Co, and Pb in urine were positively correlated with CKM risk. We then further explored the interactions between the metal mixtures (Figure 2D). The results indicated potential interactions between Cd and Ba, Cd and Cs, Cd and Pb, Co and Cs, as well as Co and Pb on CKM risk.

### 3.4 Mediation analyses

Table 3 presents the correlation between TyG, WWI, eGFR, and CKM risk. For each 1-unit increase in TyG, the risk of CKM increases by 69% (95% CI: 1.45, 1.96). Similarly, each 1-unit increase in WWI is associated with an increased risk of CKM (OR: 1.04, 95% CI: 1.32, 2.13). Furthermore, for each 1-unit increase in eGFR, the risk of CKM decreases by 3% (95% CI: 0.96, 0.98).

**TABLE 3:** Association between TyG, WWI, eGFR, and CKM.

Variables	OR	95% CI	*p*-Value
TyG – CKM	1.69	(1.45, 1.96)	<0.001
WWI – CKM	1.68	(1.32, 2.13)	<0.001
eGFR – CKM	0.97	(0.96, 0.98)	<0.001

Adjusted for age, sex, education level, marital status, PIR, race, obesity, smoking, drinking, hypertension, diabetes, and hyperlipidemia. TyG, triglyceride-glucose index; WWI, weight-adjusted waist index; eGFR, estimated glomerular filtration rate; CKM, cardiovascular-kidney-metabolic syndrome.

Table 4 shows the association between urinary heavy metals and TyG, WWI, and eGFR. The results indicate that urinary Cd, Co, Cs, Pb, and Tl are negatively correlated with TyG (*p* < 0.001). Cd, Co, Cs, Mo, and Pb are positively correlated with an increase in WWI (*p* < 0.001). Additionally, Ba, Cd, Mo, Sb, and Tl are positively correlated with eGFR, while Cs and Pb are negatively correlated with eGFR (*p* < 0.05).

**TABLE 4:** Association between urinary metals, TyG, WWI, and eGFR.

Urine metals (log-μg/g creatinine)	TyG [β (95% CI)]^a^	*p*-Value	WWI [β (95% CI)]^a^	*p*-Value	eGFR [β (95% CI)]^a^	*p*-Value
Ba	0.040 (0.010, 0.070)	0.005	0.050 (0.020, 0.080)	0.001	3.640 (2.880, 4.410)	<0.001
Cd	0.060 (0.030, 0.080)	<0.001	0.080 (0.040, 0.110)	<0.001	1.460 (0.510, 2.410)	0.003
Co	−0.070 (−0.120, −0.030)	<0.001	0.200 (0.150, 0.250)	<0.001	0.560 (−0.480, 1.600)	0.29
Cs	−0.080 (−0.130, −0.040)	<0.001	0.220 (0.140, 0.300)	<0.001	−1.900 (−3.180, −0.610)	0.004
Mo	−0.005 (−0.051, 0.042)	0.848	0.120 (0.070, 0.160)	<0.001	2.640 (1.450, 3.830)	<0.001
Pb	0.060 (0.030, 0.090)	<0.001	0.140 (0.100, 0.180)	<0.001	−4.210 (−5.220, −3.200)	<0.001
Sb	−0.010 (−0.040, 0.020)	0.57	0.010 (−0.030, 0.050)	0.54	1.996 (0.816, 3.175)	0.001
Tl	−0.110 (−0.160, −0.070)	<0.001	−0.001 (−0.055, 0.053)	0.97	7.153 (5.798, 8.507)	<0.001
W	0.010 (−0.016, 0.036)	0.459	0.037 (0.006, 0.068)	0.021	0.927 (−0.033, 1.888)	0.17

*^a^*The model was adjusted by age, sex, education level, marital status, PIR, race, obesity, smoking, drinking, hypertension, diabetes, and hyperlipidemia.

**FIGURE 3:**
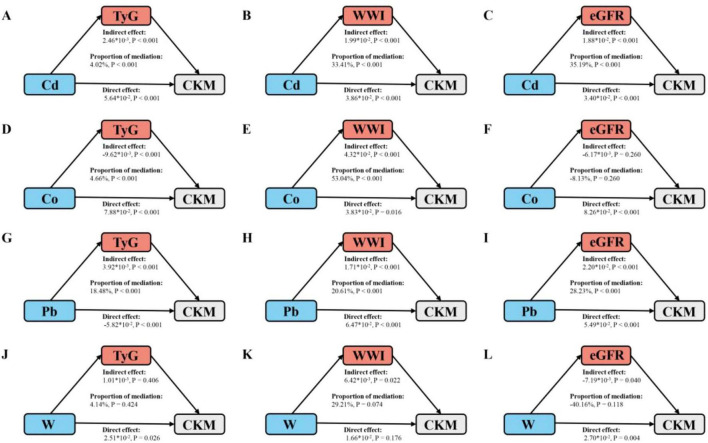
Schematic diagram of the mediation effect analysis. **(A)** TyG mediates the association between Cd exposure and CKM outcomes. **(B)** WWI mediates the association between Cd exposure and CKM outcomes. **(C)** eGFR mediates the association between Cd exposure and CKM outcomes. **(D)** TyG mediates the association between Co exposure and CKM outcomes. **(E)** WWI mediates the association between Co exposure and CKM outcomes. **(F)** eGFR mediates the association between Co exposure and CKM outcomes. **(G)** TyG mediates the association between Pb exposure and CKM outcomes. **(H)** WWI mediates the association between Pb exposure and CKM outcomes. **(I)** eGFR mediates the association between Pb exposure and CKM outcomes. **(J)** TyG mediates the association between W exposure and CKM outcomes. **(K)** WWI mediates the association between W exposure and CKM outcomes. **(L)** eGFR mediates the association between W exposure and CKM outcomes. The mediated proportion is calculated as indirect effect/ (indirect effect + direct effect) ×100%. The “*” represents multiplication. Cd, cadmium; Co, cobalt; Pb, lead; W, tungsten; TyG, triglyceride-glucose index; WWI, weight-adjusted waist index; eGFR1, estimated glomerular filtration rate; CKM, cardiovascular-kidney-metabolic syndrome. All metals were Ln-transformed before analysis. Analyses were adjusted for age, sex, education level, marital status, PIR, race, obesity, smoking, drinking, hypertension, diabetes, and hyperlipidemia.

Subsequently, mediation analysis was conducted to evaluate the potential mediating roles of TyG, WWI, and eGFR in the relationship between urinary metals and CKM prevalence. As shown in Figure 3, TyG significantly mediated the association between Cd, Co, Pb, and CKM prevalence, with mediation proportions of 4.02%, 4.66%, and 18.48%, respectively (all *p* < 0.001). WWI mediated the relationship between Cd, Co, Pb, and CKM risk, with mediation proportions of 33.41%, 53.04%, and 20.61%, respectively. Additionally, eGFR mediated the association between Cd, Pb, and CKM risk, with mediation proportions of 35.19% and 28.23%, respectively.

### 3.5 Sensitivity analysis

To evaluate model robustness, we assessed the stability of the results by increasing the number of bootstrap iterations in both the WQS and Qgcomp analyses from 10,000 to 20,000. This adjustment did not alter the findings. The ranking of heavy metals in the WQS model remained unchanged, and the direction (positive or negative) of variable weights in the Qgcomp model was also consistent with the original results (Supplementary Figures 7, 8). These findings indicate that the results are robust to changes in the number of bootstrap iterations.

## 4 Discussion

In this cross-sectional study involving 5,221 adults, we found that both single and mixed exposure models of urinary heavy metals were significantly associated with an increased prevalence of advanced CKM. Among the metals analyzed, Cd, Co, and Pb were the most dominant, with Co contributing the most. RCS analysis revealed a linear positive association between urinary Cd, Co, Pb, and W concentrations and advanced CKM prevalence, with no clear thresholds or inflection points identified within the observed exposure ranges (Supplementary Figure 2). Similarly, BKMR exposure-response curves (Figure 2C) confirmed a consistent positive relationship for Cd, Co, and Pb, suggesting that CKM risk increases progressively with metal exposure without a distinct threshold. This linearity underscores the importance of minimizing even low-level exposures to these metals to reduce CKM risk. Furthermore, TyG, WWI, and eGFR were identified as partial mediators of the association between urinary heavy metals and advanced CKM.

The primary components of CKM syndrome include obesity, CKD, CVD, diabetes, and hypertension. Studies have shown that 26.3% of people in the United States suffer from at least one of these conditions (CVD, kidney disease, or metabolic disorders), 8.0% suffer from at least two of these conditions, and 1.5% are afflicted with all three (23). In China, the prevalence rates of CVD, diabetes, and CKD are 23.4%, 11.2%, and 10.8%, respectively, and these rates continue to rise (24–26). Additionally, CVD, diabetes, and CKD are recognized as major causes of mortality (27). Heavy metal pollution is an environmental issue that cannot be overlooked in modern society. It not only causes cytotoxicity, damages bodily systems such as the nervous and hematopoietic systems, but also severely impacts the kidneys’ ability to excrete waste and toxins, leading to irreversible harm to human health. The concentration of metals in the blood provides real-time information regarding metal exposure, distribution, and transport within the body, while the metal content in urine reflects cumulative exposure and excretion. Considering the sample size, this study selected urine samples for heavy metal analysis. Previous studies have mainly focused on the association between heavy metal exposure and individual diseases (6, 28, 29) (6/7/8). Our study, however, links cardiovascular, kidney, and metabolic pathology, providing, for the first time, epidemiological evidence of a significant positive correlation between urinary heavy metals and the risk of advanced CKM. It is noteworthy that heavy metals in the environment typically exist in a co-existing form, and this mixed exposure pattern may lead to a more severe disease burden. For instance, Shi et al. (30) found interactions between nine pairs of heavy metals in CKD patients, and compared to single exposures, mixed exposure exacerbated the risk of CKD. In this study, we used WQS, qpcomp, and BKMR models to comprehensively evaluate the impact of mixed heavy metal exposure on advanced CKM from the perspectives of overall effect, positive and negative effect components, interactions, and non-linear effects. The WQS results indicate that Co is the strongest risk factor for advanced CKM. Both qpcomp and BKMR models confirmed this finding and demonstrated that mixed metal exposure is positively correlated with advanced CKM risk, further suggesting that metal exposure may promote the progression of advanced CKM.

Cobalt is a transition metal widely used in battery manufacturing, alloy production, industrial pigments, radiation therapy, and medical device sterilization. However, excessive exposure can be harmful to human health. Five cobalt compounds and metallic cobalt itself have been classified as Class 1 sensitizers. Animal studies have shown that direct contact with cobalt or its compounds may induce allergic dermatitis. Epidemiological research indicates that long-term inhalation of cobalt dust or fumes can lead to “cobalt lung” (31), and workers exposed to cobalt are at a higher risk of developing asthma (32). Our study shows that Co is the strongest risk factor for advanced CKM, and the underlying mechanisms may involve the following: once cobalt enters the body, it is converted to Co^2+^ through ion dissolution, reduction, and intracellular metabolism (33). When Co^2+^ enter the bloodstream and lymphatic circulation, and subsequently diffuse to various organs, they can induce oxidative stress, mitochondrial dysfunction, and cell apoptosis through multiple pathways, leading to cardiovascular and renal dysfunction (34).

Cadmium and Pb, as ubiquitous environmental pollutants, are associated with various exposure sources, including environmental contamination, industrial production, and lifestyle habits. Both metals can enter the human body through inhalation, ingestion, and, to a lesser extent, skin contact. Their widespread presence in the environment and bioaccumulation through the food chain make them significant public health concerns. Although their metabolic and accumulation patterns in the body differ, both are recognized as risk factors for CVD, CKD, and metabolic-related diseases. Firstly, both Pb and Cd are well-established nephrotoxins (35, 36). Cd directly damages renal tubular epithelial cells, impairing reabsorption and excretion functions, while lead induces damage to both the glomeruli and renal tubules. This kidney damage disrupts the kidneys’ ability to regulate blood pressure, electrolyte balance, and metabolic homeostasis. Secondly, both metals are strong inducers of oxidative stress and inflammation (37). They promote the production of reactive oxygen species (ROS) and pro-inflammatory cytokines such as TNF-α, IL-6, and IL-1β, which can damage cellular components such as lipids, proteins, and DNA. Lastly, Cd and Pb contribute to metabolic disturbances, ultimately leading to a vicious cycle of insulin resistance, dyslipidemia, and inflammation (38, 39). Renal damage, oxidative stress, inflammation, and metabolic dysfunction are all major driving factors in the pathogenesis of advanced CKM. Existing studies also support our findings, with Pan et al. (40) observing that high concentrations of Pb, Cd, and Co in combination pose a significant risk for CKD. These findings underscore the importance of reducing heavy metal exposure, strengthening environmental regulation, and raising public health awareness as critical measures to protect human health.

Given the toxic effects of heavy metal exposure on the cardiovascular system, kidneys, and metabolism, we selected TyG, WWI, and eGFR as mediators for analysis. TyG, as a marker of insulin resistance, integrates the effects of heavy metal exposure on glucose and lipid metabolism. Metals like Cd and Pb can induce oxidative stress and activate pathways such as MAPK and PI3K/Akt, impairing glucose uptake in peripheral tissues and promoting insulin resistance (41). This leads to elevated TyG, which exacerbates dyslipidemia, hypertension, and chronic inflammation, key drivers of CKM (42). Insulin resistance may also directly affect cardiovascular and kidney function by activating the renin-angiotensin-aldosterone system (RAAS) and the sympathetic nervous system (43). WWI, reflecting visceral adiposity, captures the impact of metals on adipose tissue dysfunction. Heavy metals may disrupt adipocyte differentiation and function, increasing secretion of pro-inflammatory cytokines (e.g., IL-6 and TNF-α) and reducing adiponectin, which amplifies systemic inflammation and atherosclerosis (44). These processes link environmental exposures to CKM through metabolic and inflammatory pathways, with visceral fat acting as both a storage site for metals and an endocrine organ exacerbating cardiometabolic risk. Under the context of heavy metal exposure, these factors may further exacerbate systemic inflammation and oxidative stress, thereby promoting atherosclerosis, insulin resistance, and kidney fibrosis (45). Additionally, central obesity is closely associated with lipid metabolism disorders, hypertension, and abnormal glucose metabolism, all of which together form the core pathophysiological basis of CKM syndrome. Finally, eGFR is a key indicator for evaluating kidney function. Heavy metals can directly damage the renal tubules and glomeruli, leading to a decline in the glomerular filtration rate. A reduction in eGFR not only reflects kidney damage but may also affect systemic metabolism and cardiovascular health through various mechanisms. For instance, impaired kidney function leads to the accumulation of uremic toxins (such as urea and creatinine) and metabolic waste products, further exacerbating oxidative stress and inflammatory responses (46). Additionally, renal insufficiency can affect the activation of RAAS and electrolyte balance, increasing the risk of hypertension and CVDs (47). Moreover, kidney damage may indirectly influence bone health and anemia by reducing the synthesis of active vitamin D and erythropoietin secretion, thereby establishing a vicious cycle (48, 49). The three mediators—TyG, WWI, and eGFR—are interconnected and complementary. Insulin resistance (reflected by TyG) promotes central obesity (reflected by WWI), which in turn exacerbates renal dysfunction (reflected by eGFR). Conversely, kidney damage further worsens insulin resistance and metabolic dysregulation. Heavy metal exposure amplifies this interaction, creating a synergistic effect that accelerates the progression of CKD.

The negative association between certain metals (e.g., Co, Cs, and Tl) and TyG, alongside TyG’s mediating role in CKM risk, may seem counterintuitive. However, this likely reflects the complex metabolic effects of heavy metals, which may lower TyG through mechanisms such as disrupted lipid metabolism or oxidative stress, while still contributing to CKM risk via other pathways. The small mediation proportion for Co (4.66%) suggests that TyG is one of several pathways linking metal exposure to CKM, with other mechanisms, such as inflammation or direct organ toxicity, likely playing larger roles.

Our study offers several notable strengths. First, it is the first to explore the association between heavy metal exposure and CKM syndrome. Moreover, we employed multiple statistical approaches and adjusted for potential confounders, which strengthens the robustness and reliability of our findings. However, there are also some limitations. The primary constraint is the cross-sectional nature of the NHANES design, which prevents the establishment of causal relationships. While our mediation analysis suggests that TyG, WWI, and eGFR may partially explain the association between heavy metal exposure and advanced CKM, these findings should be interpreted cautiously due to the cross-sectional nature of the study, which precludes establishing causality. Longitudinal studies are needed to confirm these mediating pathways. A key limitation of this study is the use of urinary metal concentrations, which primarily reflect recent exposure rather than long-term or cumulative burden, as blood or tissue levels might provide. Future studies incorporating biomarkers of cumulative exposure, such as blood or bone lead levels, could provide a more comprehensive understanding of long-term metal toxicity in CKM. Additionally, the study population is largely composed of U.S. adults, which may limit the generalizability of the results to other populations with different genetic, lifestyle, or environmental factors. Finally, the assessment of heavy metal exposure at a single time point may not accurately capture long-term or cumulative exposure.

The exclusion of 64,969 NHANES participants due to missing data on age, pregnancy, metals, or mediators may introduce selection bias, potentially limiting generalizability. Comparison of included and excluded participants showed similar race and socioeconomic distributions, but excluded individuals were slightly younger and more likely female, suggesting possible bias toward healthier participants with complete data. This could underestimate the association between heavy metal exposure and CKM risk. Future studies with imputation methods or broader inclusion criteria could enhance generalizability.

## 5 Conclusion

Our findings highlight the need for public health strategies to reduce heavy metal exposure and CKM risk. Key interventions include strengthening environmental regulations to limit Cd, Co, and Pb contamination, enforcing stricter occupational exposure limits, raising public awareness of exposure sources, and implementing biomonitoring and early screening for TyG, WWI, and eGFR in high-risk populations. These measures, alongside further research, could mitigate the public health burden of CKM syndrome.

## Data Availability

The original contributions presented in the study are included in the article/Supplementary material, further inquiries can be directed to the corresponding authors.
