# Supplementary material for: Association between urinary heavy metals and cardiovascular-kidney-metabolic syndrome: mediating roles of TyG, WWI, and eGFR
Source: Front Nutr. 2025 Jun 25;12:1613721. doi: 10.3389/fnut.2025.1613721 (PMC12237628; doi:10.3389/fnut.2025.1613721)
Supplement: Supplementary file 1 [file Data_Sheet_1.docx]

**Supplementary Material**

Table S1. Definitions of CKM Syndrome Stages

| **CKM Syndrome Stages** | **Definition** |
| --- | --- |
| Stage 0: No CKM risk factors | Individuals meet all following criteria.   1. Not overweight/obese (body mass index <25 kg/m^2^, or <23 kg/m^2^ if Asian ancestry; Waist circumference ≥88/102 cm in women/men (or if Asian ancestry, ≥80/90 cm in women/men) 2. Without metabolic risk factors (hypertension, hypertriglyceridemia [<135 mg/dL], metabolic syndrome, prediabetes, diabetes) or chronic kidney disease (CKD). |
| Stage 1: Excess or dysfunctional adiposity | Individuals with body mass index ≥25 kg/m^2^ (or ≥ 23 kg/m^2^ if Asian ancestry), waist circumference ≥88/102 cm in women/men (or if Asian ancestry, ≥80/90 cm in women/men), and/or prediabetes without the presence of other metabolic risk factors or CKD. |
| Stage 2: Metabolic risk factors and CKD | Individuals with metabolic risk factors (hypertriglyceridemia [≥135 mg/dL], hypertension, metabolic syndrome, diabetes), or CKD. |
| Stage 3: Subclinical CVD in CKM | Individuals with very high-risk CKD or high predicted 10-year CVD risk (≥ 20% using PREVENT [Predict Risk of cardiovascular disease EVENTs] base model |
| Stage 4: Clinical CVD in CKM | Individuals with clinical CVD (coronary heart disease, myocardial infarction, stroke or peripheral artery disease) |

CKM, cardiovascular-kidney-metabolic; CKD, chronic kidney disease; CVD, cardiovascular disease.

**Table S2.** Definition of variables involved in this study.

| Variables | Description in NHANES |
| --- | --- |
| Age | Divided into three groups: 20-40 years old, 41-60  years old, >60 years old |
| Sex | Male and Female |
| Race | Mexican American, Non-Hispanic Black, Non-Hispanic White, Other Race |
| Educational level | Below high school, High School or above |
| Marital status | Yes: Married/Living with partner |
| PIR | Poor: <1.3; Not Poor:>=1.3 |
| Obesity | Yes: BMI>=30 |
| Smoking | Smoking status was grouped into never smoker (defined as <100 cigarettes in a lifetime), current smoker (defined as ≥100 cigarettes in a lifetime), and former smoker (defined as ≥100 cigarettes and had quit smoking) |
| Drinking | heavy drinking (≥4 drinks/day for men, ≥3 drinks/day for women, or ≥5 days of drinking in a month),  moderate drinking (≥3 drinks/day for men, ≥2 drinks/day for women, or ≥2 days of drinking in a month),  mild drinking (≤2 drinks/day for men, ≤1 drink/day for women, and ≥12 drinks in a year),  and never-drinking (total number of drinks in a year <12, and dietary alcohol content of 0%) |
| Diabetes | Diabetes was defined as a history of previous diabetes, HbA1c level ≥6.5%, or fasting blood glucose level ≥126 mg/dL |
| Hypertension | The diagnostic criteria consist of self-reported hypertension history, the utilization of antihypertensive medication, a systolic blood pressure (SBP)  ≥ 140mmHg, or a diastolic blood pressure (DBP)  ≥ 90mmHg |
| Hyperlipidemia | (1) Triglyceride (TG) levels ≥150 mg/dl (1.7 mmol/L); (2) Total cholesterol (TC) levels ≥200 mg/dl (5.18 mmol/L); (3) Low-density lipoprotein (LDL) levels ≥130 mg/dl (3.37 mmol/L); (4) High-density lipoprotein (HDL) levels: Men: <40 mg/dl (1.04 mmol/L); Women: <50 mg/dl (1.30 mmol/L); (5) Individuals taking cholesterol-lowering drugs are also considered hyperlipidemia. |

PIR, Ratio of family income to poverty.

**Table S3.** Baseline characteristics of all participants were stratified by CKM stages, weighted.

| **Characteristic** | **Overall**, N = 31,065,216 (100%) | **CKM 0**, N = 3,520,503 (11%) | **CKM 1**, N = 6,952,938 (22%) | **CKM 2**, N = 16,913,617 (55%) | **CKM 3**, N = 1,126,545 (3.6%) | **CKM 4**, N = 2,551,613 (8.4%) | **P Value** |
| --- | --- | --- | --- | --- | --- | --- | --- |
| **No. of participants in the sample** | 5,221 | 473 | 1,025 | 2,842 | 315 | 566 | **-** |
| **Age (%)** |  |  |  |  |  |  | **<0.001** |
| *20-40* | 11,690,744 (38%) | 2,319,974 (66%) | 3,751,975 (54%) | 5,471,698 (32%) | 2,876 (0.3%) | 144,220 (5.7%) |  |
| *41-60* | 11,974,829 (39%) | 1,055,033 (30%) | 2,436,672 (35%) | 7,717,698 (46%) | 69,021 (6.1%) | 696,405 (27%) |  |
| *>60* | 7,399,643 (24%) | 145,496 (4.1%) | 764,291 (11%) | 3,724,222 (22%) | 1,054,648 (94%) | 1,710,987 (67%) |  |
| **Sex (%)** |  |  |  |  |  |  | **<0.001** |
| *Female* | 15,410,293 (50%) | 2,247,742 (64%) | 3,631,505 (52%) | 8,047,981 (48%) | 421,380 (37%) | 1,061,685 (42%) |  |
| *Male* | 15,654,923 (50%) | 1,272,761 (36%) | 3,321,433 (48%) | 8,865,637 (52%) | 705,165 (63%) | 1,489,928 (58%) |  |
| **Race (%)** |  |  |  |  |  |  | **<0.001** |
| *Non-Hispanic White* | 20,980,865 (68%) | 2,589,978 (74%) | 4,589,541 (66%) | 11,080,410 (66%) | 833,174 (74%) | 1,887,763 (74%) |  |
| *Other* | 4,042,763 (13%) | 501,463 (14%) | 882,159 (13%) | 2,319,550 (14%) | 110,358 (9.8%) | 229,232 (9.0%) |  |
| *Non-Hispanic Black* | 3,217,873 (10%) | 219,891 (6.2%) | 703,602 (10%) | 1,879,066 (11%) | 115,474 (10%) | 299,841 (12%) |  |
| *Mexican American* | 2,823,714 (9.1%) | 209,171 (5.9%) | 777,636 (11%) | 1,634,592 (9.7%) | 67,539 (6.0%) | 134,776 (5.3%) |  |
| **Married/live with partner (%)** |  |  |  |  |  |  | 0.101 |
| *no* | 10,948,604 (35%) | 1,394,979 (40%) | 2,346,587 (34%) | 5,801,504 (34%) | 477,910 (42%) | 927,624 (36%) |  |
| *yes* | 20,112,208 (65%) | 2,125,524 (60%) | 4,606,351 (66%) | 11,109,127 (66%) | 647,216 (58%) | 1,623,989 (64%) |  |
| **Education level (%)** |  |  |  |  |  |  | **<0.001** |
| *Below high school* | 5,159,568 (17%) | 365,091 (10%) | 830,768 (12%) | 2,980,113 (18%) | 320,223 (29%) | 663,375 (26%) |  |
| *High School or above* | 25,899,476 (83%) | 3,155,412 (90%) | 6,122,170 (88%) | 13,931,799 (82%) | 803,304 (71%) | 1,886,790 (74%) |  |
| **PIR (%)** |  |  |  |  |  |  | **<0.001** |
| *Poor* | 6,033,864 (21%) | 625,952 (19%) | 1,153,623 (18%) | 3,268,172 (21%) | 324,296 (32%) | 661,820 (28%) |  |
| *Not Poor* | 22,987,914 (79%) | 2,661,696 (81%) | 5,386,626 (82%) | 12,532,815 (79%) | 688,315 (68%) | 1,718,462 (72%) |  |
| **Obesity (%)** |  |  |  |  |  |  | **<0.001** |
| *no* | 19,902,198 (64%) | 3,520,503 (100%) | 5,071,480 (73%) | 9,084,340 (54%) | 701,983 (62%) | 1,523,892 (60%) |  |
| *yes* | 11,146,166 (36%) | 0 (0%) | 1,870,889 (27%) | 7,826,155 (46%) | 421,401 (38%) | 1,027,721 (40%) |  |
| **Smoking (%)** |  |  |  |  |  |  | **<0.001** |
| *never* | 16,868,950 (54%) | 2,231,117 (63%) | 4,065,891 (58%) | 9,124,161 (54%) | 476,501 (42%) | 971,280 (38%) |  |
| *former* | 8,002,121 (26%) | 547,848 (16%) | 1,804,589 (26%) | 4,064,443 (24%) | 487,649 (43%) | 1,097,592 (43%) |  |
| *current* | 6,191,046 (20%) | 741,538 (21%) | 1,080,361 (16%) | 3,724,012 (22%) | 162,394 (14%) | 482,741 (19%) |  |
| **Drinking (%)** |  |  |  |  |  |  | **<0.001** |
| *never* | 2,905,745 (10%) | 289,278 (9.1%) | 504,379 (7.8%) | 1,645,920 (11%) | 199,953 (20%) | 266,216 (11%) |  |
| *former* | 3,525,297 (12%) | 184,087 (5.8%) | 580,893 (9.0%) | 1,937,389 (12%) | 258,182 (26%) | 564,746 (24%) |  |
| *mild* | 10,917,403 (38%) | 1,132,637 (36%) | 2,370,689 (37%) | 6,112,409 (39%) | 412,304 (42%) | 889,364 (38%) |  |
| *moderate* | 5,031,520 (18%) | 812,525 (25%) | 1,405,585 (22%) | 2,511,683 (16%) | 29,608 (3.0%) | 272,118 (12%) |  |
| *heavy* | 6,227,108 (22%) | 771,211 (24%) | 1,575,830 (24%) | 3,467,796 (22%) | 87,364 (8.8%) | 324,907 (14%) |  |
| **Hypertension (%)** |  |  |  |  |  |  | **<0.001** |
| *no* | 19,640,523 (63%) | 3,520,503 (100%) | 6,952,938 (100%) | 8,141,731 (48%) | 300,894 (27%) | 724,457 (28%) |  |
| *yes* | 11,424,693 (37%) | 0 (0%) | 0 (0%) | 8,771,887 (52%) | 825,651 (73%) | 1,827,156 (72%) |  |
| **Diabetes (%)** |  |  |  |  |  |  | **<0.001** |
| *no* | 26,119,834 (84%) | 3,520,503 (100%) | 6,952,938 (100%) | 13,521,445 (80%) | 516,992 (46%) | 1,607,956 (63%) |  |
| *yes* | 4,945,382 (16%) | 0 (0%) | 0 (0%) | 3,392,173 (20%) | 609,553 (54%) | 943,657 (37%) |  |
| **Hyperlipidemia (%)** |  |  |  |  |  |  | **<0.001** |
| *no* | 9,369,135 (30%) | 2,158,792 (61%) | 3,426,558 (49%) | 3,258,702 (19%) | 269,870 (24%) | 255,212 (10%) |  |
| *yes* | 21,696,081 (70%) | 1,361,711 (39%) | 3,526,381 (51%) | 13,654,915 (81%) | 856,674 (76%) | 2,296,400 (90%) |  |
| **TyG (mean (SD))** | 8.58 (0.67) | 7.98 (0.39) | 8.20 (0.38) | 8.82 (0.65) | 8.88 (0.72) | 8.77 (0.72) | **<0.001** |
| **WWI (mean (SD))** | 10.94 (0.81) | 10.14 (0.54) | 10.69 (0.73) | 11.08 (0.76) | 11.68 (0.67) | 11.46 (0.76) | **<0.001** |
| **eGFR (mean (SD))** | 95.89 (21.91) | 105.51 (18.53) | 102.23 (18.64) | 96.12 (20.10) | 66.03 (21.14) | 76.95 (23.75) | **<0.001** |

Mean (SD) for continuous variables: the P value was calculated by the weighted One-Way ANOVA.

Percentages (weighted N, %) for categorical variables: the P value was calculated by the weighted chi-square test.

Abbreviation: TyG, Triglyceride-glucose index; WWI, Weight-adjusted waist index; eGFR, estimated glomerular filtration rate; PIR, poverty income ratio; CKM, advanced Cardiovascular-kidney-metabolic syndrome.

**Table S4.** Distribution of metal concentrations among all subjects.

|  |  | **Percentile** |  |  |  |  |
| --- | --- | --- | --- | --- | --- | --- |
| **Exposure biomarkers** | **Detection frequency** | **25^th^** | **50^th^** | **75^th^** | **GM** | **Mean** |
| Ba | 99.570% | 0.550 | 1.070 | 2.020 | 1.054 | 1.719 |
| Cd | 98.452% | 0.146 | 0.285 | 0.533 | 0.276 | 0.428 |
| Co | 99.865% | 0.216 | 0.346 | 0.533 | 0.345 | 0.511 |
| Cs | 98.776% | 2.820 | 4.540 | 6.700 | 4.243 | 5.183 |
| Mo | 99.856% | 21.800 | 39.100 | 64.110 | 36.343 | 48.710 |
| Pb | 98.353% | 0.240 | 0.440 | 0.770 | 0.430 | 0.680 |
| Sb | 80.253% | 0.029 | 0.049 | 0.083 | 0.052 | 0.077 |
| Tl | 99.388% | 0.101 | 0.165 | 0.254 | 0.156 | 0.195 |
| W | 90.556% | 0.031 | 0.062 | 0.118 | 0.063 | 0.109 |

Abbreviations: Ba, barium; Cd, cadmium; Co, cobalt; Cs, cesium; Mo, molybdenum; Pb, lead; Sb, antimony; Tl, thallium; W, tungsten; GM, geometric mean.


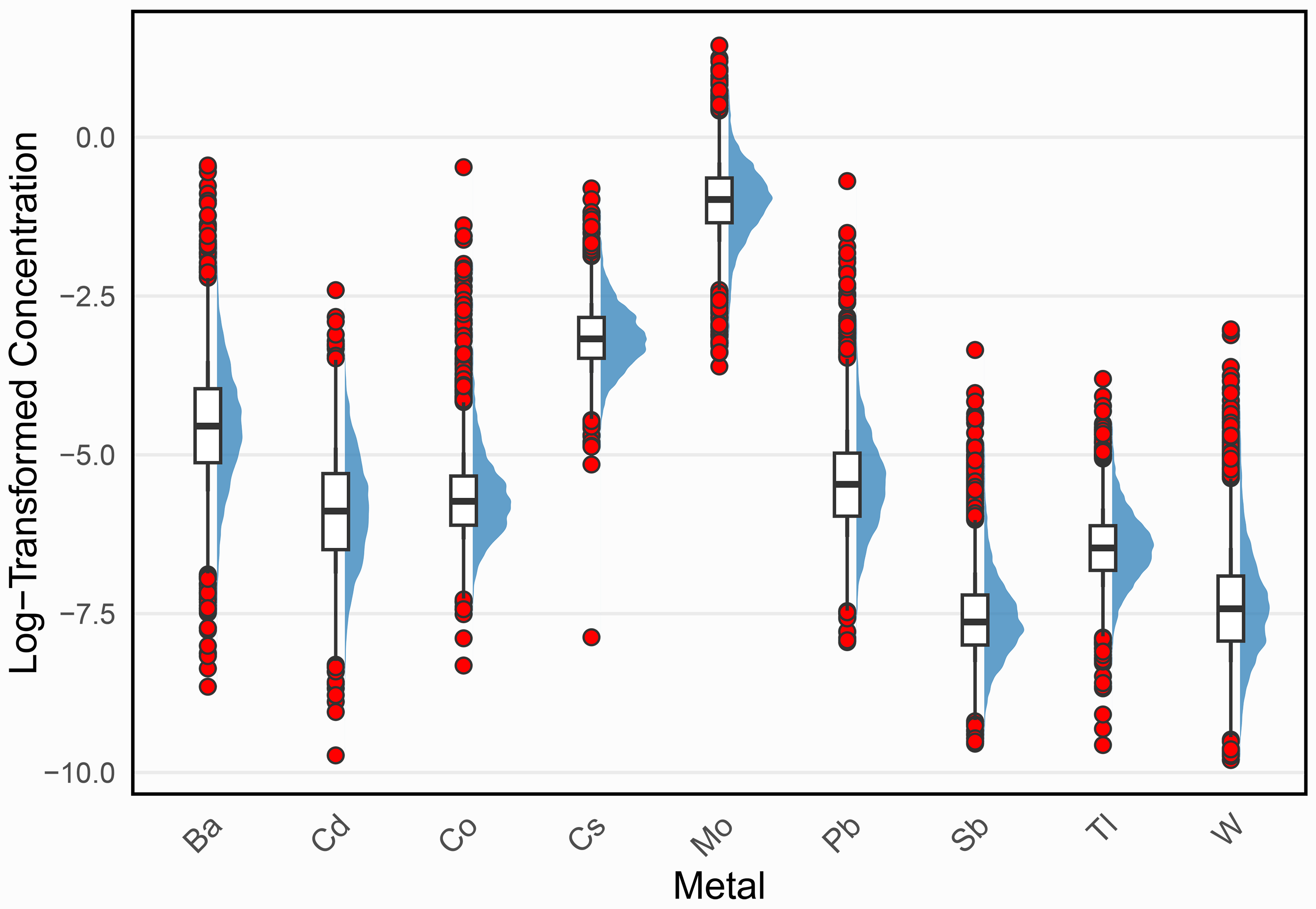


**Figure S1.** Rain cloud plots to identify outliers in log-transformed urinary metal concentrations, with red dots indicating outliers. Rain cloud plots for the nine urinary metals (Ba, Cd, Co, Cs, Mo, Pb, Sb, Tl, W), showing density curves, boxplots, and jittered points, with red dots marking outliers.


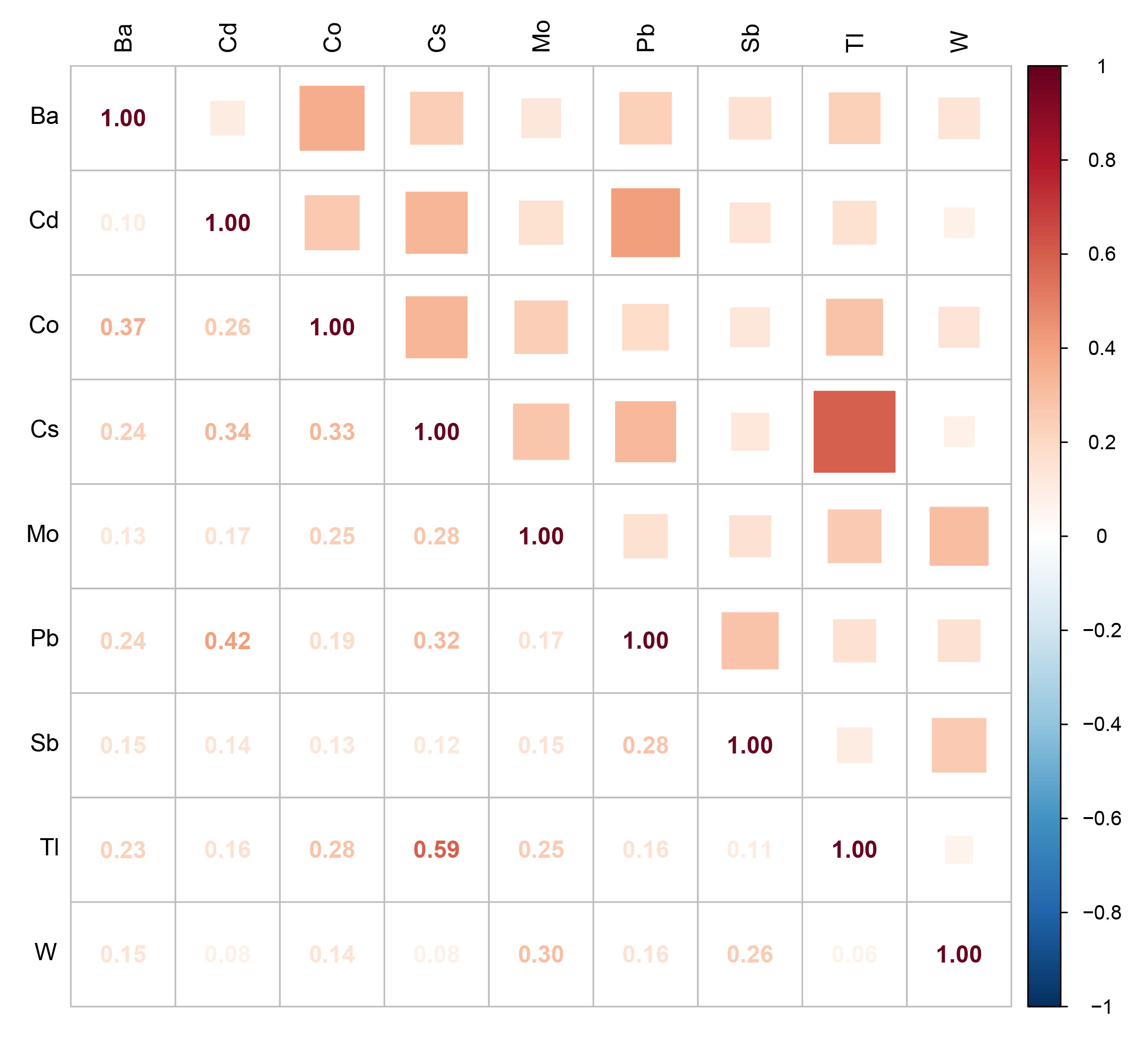


**Figure S2.** Pearson's correlation analysis between ln-transformed concentrations of metals

Abbreviations: Ba, barium; Cd, cadmium; Co, cobalt; Cs, cesium; Mo, molybdenum; Pb, lead; Sb, antimony; Tl, thallium; W, tungsten.





**Figure S3.** Dose-response relationship between metal and CKM were estimated by RCS models. All metals were ln-transformed before analysis. The model was adjusted by age, sex, education level, marital status, PIR, race, obesity, smoking, drinking, hypertension, diabetes, and hyperlipidemia.


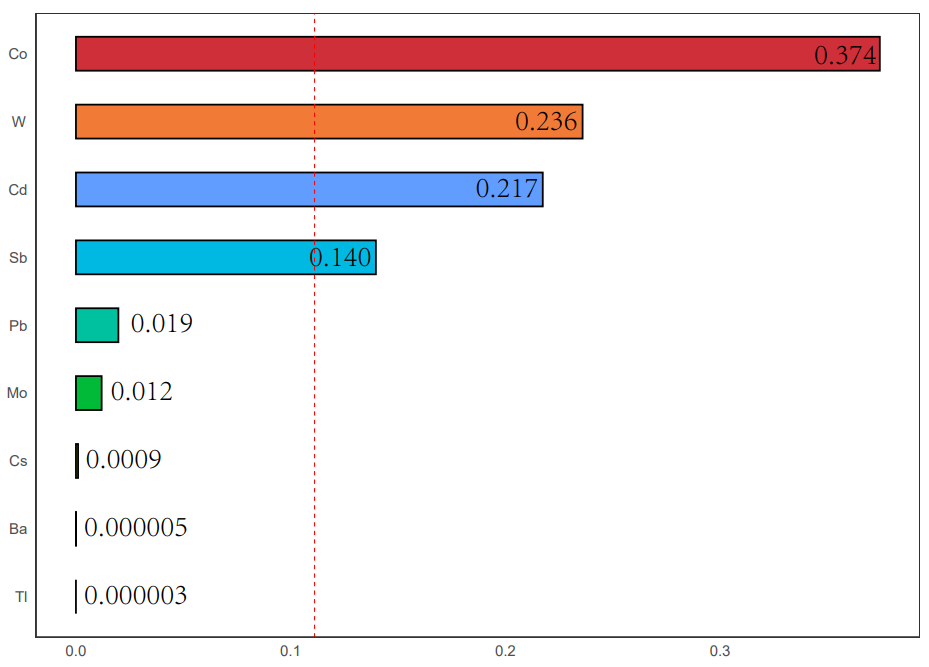


**Figure S4.** Weighted values of urinary metals for CKM in WQS models. All metals were ln-transformed before analysis. Models were adjusted for age, sex, education level, marital status, PIR, race, obesity, smoking, drinking, hypertension, diabetes, and hyperlipidemia.


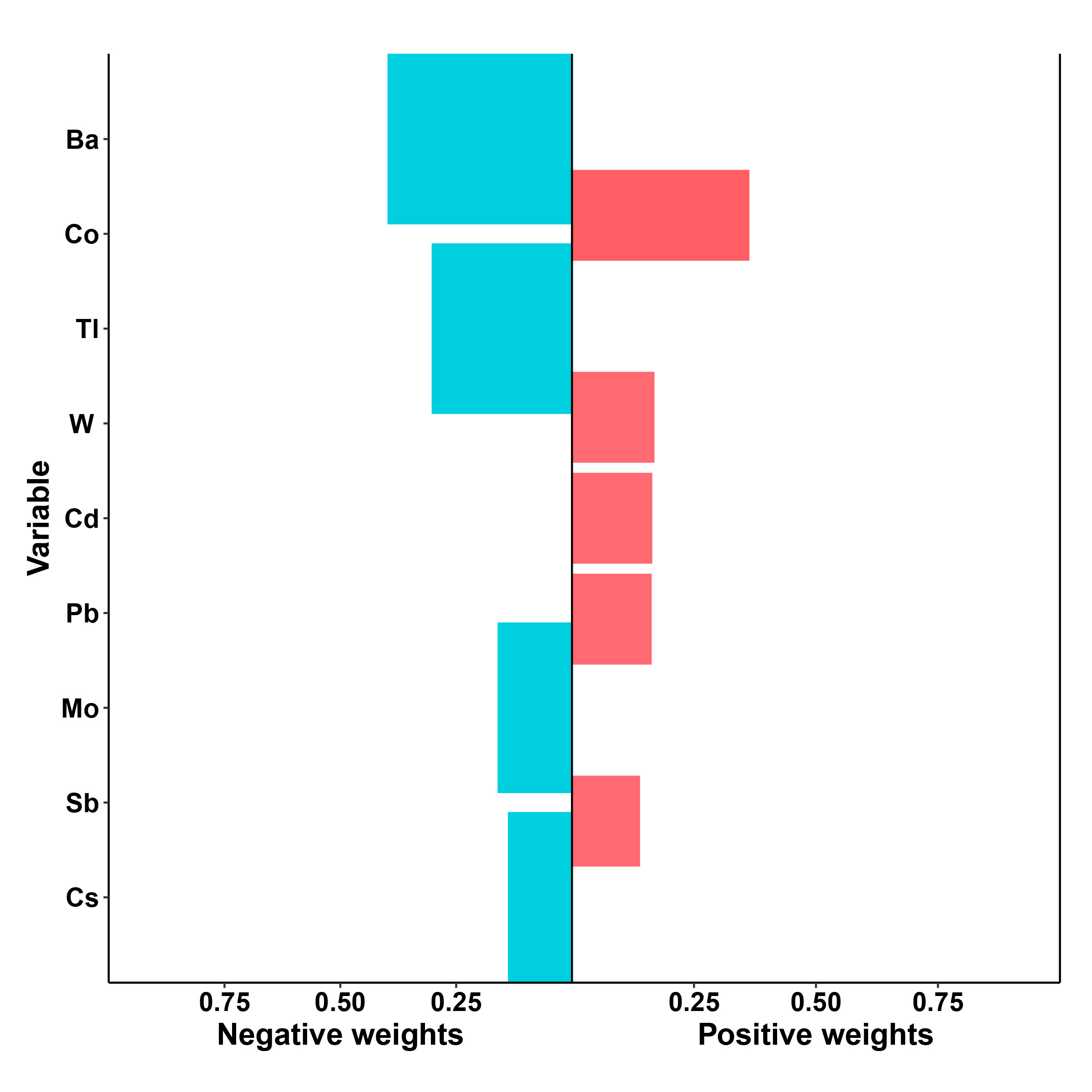


**Figure S5.** The qgcomp model weights of the urinary metals for CKM. Note: Ba, barium; Cd, cadmium; Co, cobalt; Cs, cesium; Mo, molybdenum; Pb, lead; Sb, antimony; Tl, thallium; W, tungsten. All metals were ln-transformed before analysis. The model was adjusted by age, sex, education level, marital status, PIR, race, obesity, smoking, drinking, hypertension, diabetes, and hyperlipidemia.


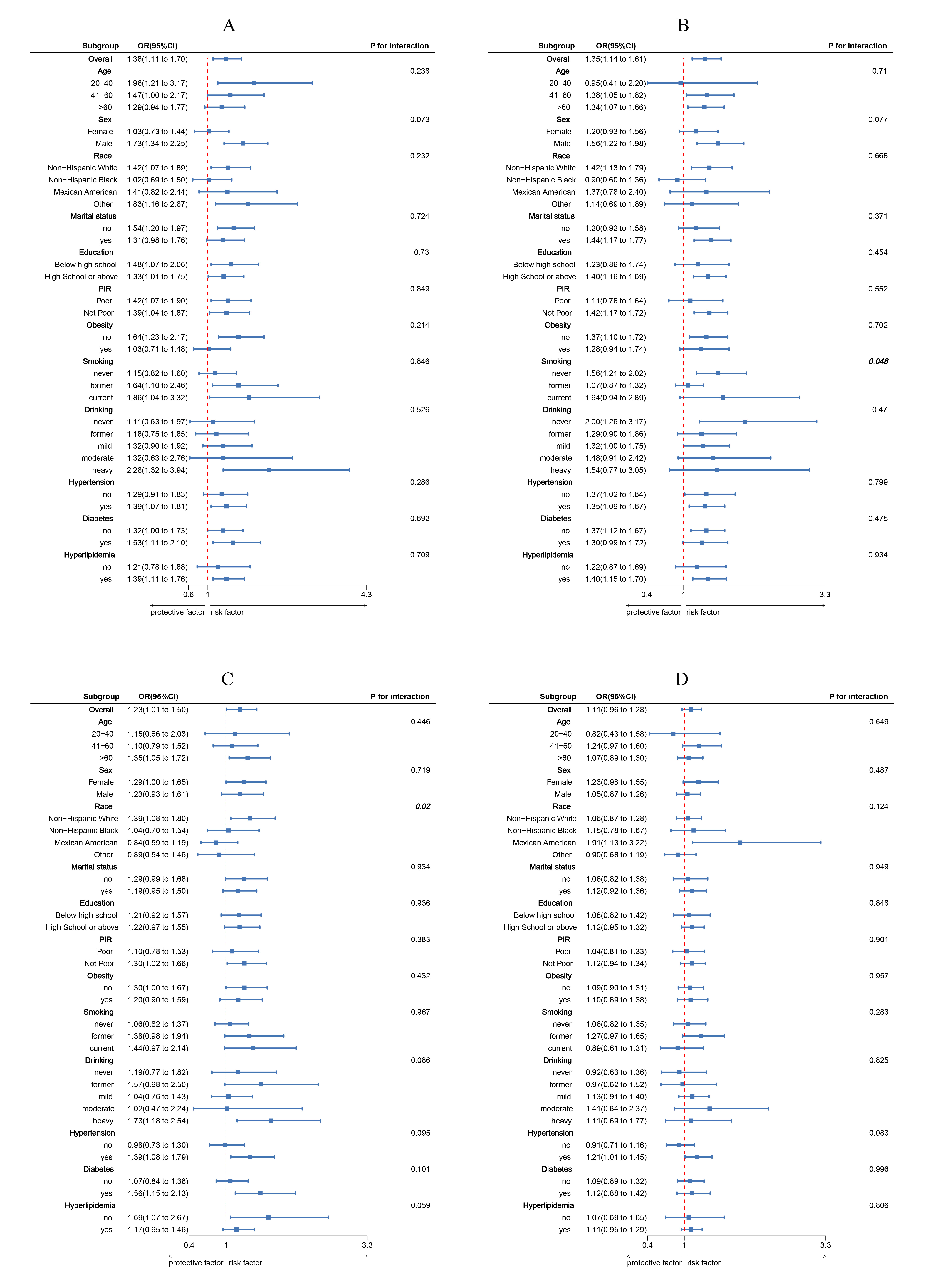


**Figure S6.** Subgroup analysis between urinary metals and CKM. A, Cd - CKM; B, Co - CKM; C, Pb - CKM; D, W - CKM. ORs were calculated each standard deviation increased in metals. All metals were ln-transformed before analysis. Analyses were adjusted for age, sex, education level, marital status, PIR, race, obesity, smoking, drinking, hypertension, diabetes, and hyperlipidemia.


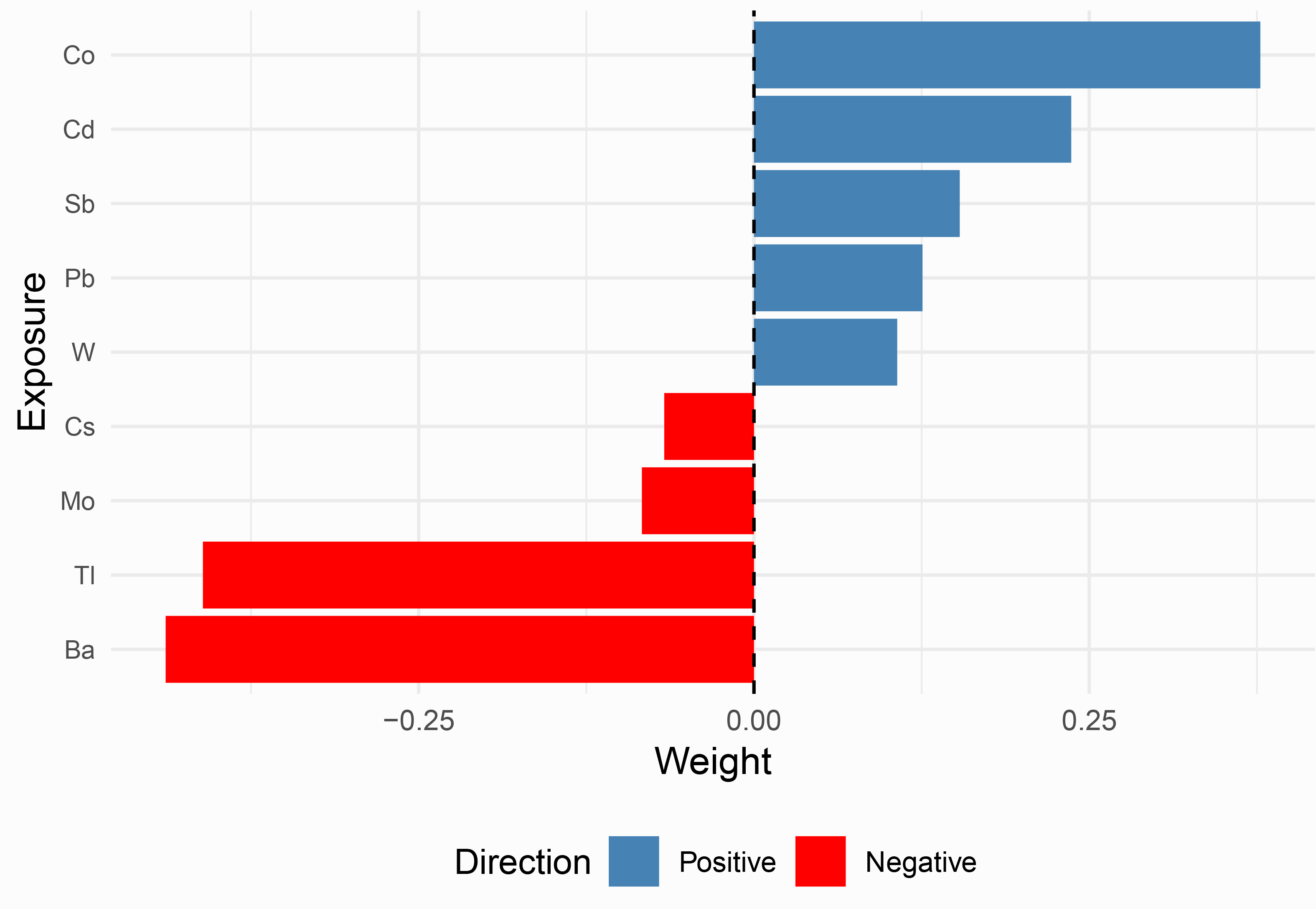


**Figure S7.** Qgcomp plot with the bootstrap test iterations increased to 20,000. Qgcomp bar plot showing mixture weights for the metals, with bootstrap iterations increased to 20,000 to test model stability.


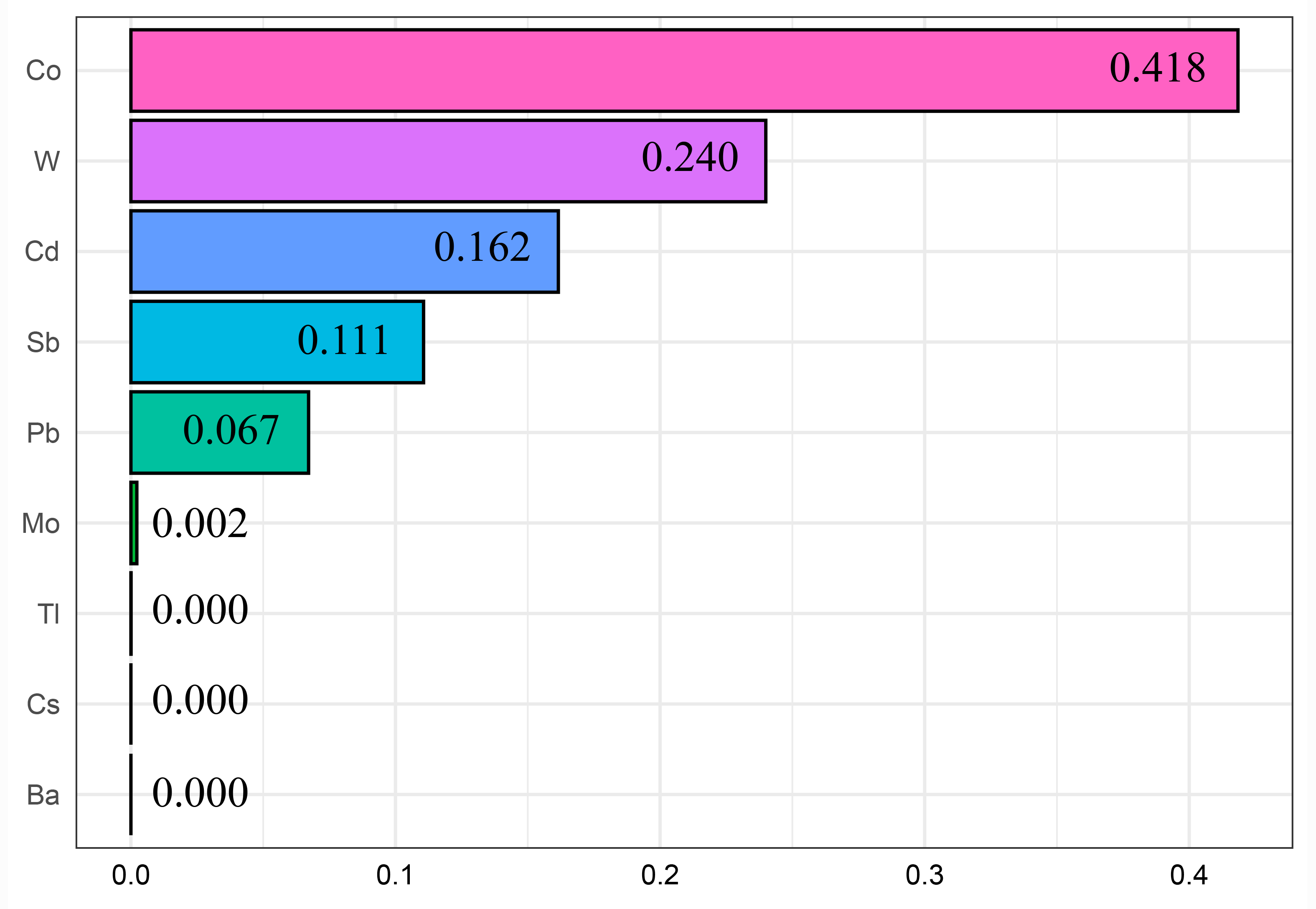


**Figure S8.** WQS plot with the bootstrap test iterations increased to 20,000. WQS bar plot showing mixture weights, with bootstrap iterations increased to 20,000.
